# Anti-diabetic effect of a preparation of vitamins, minerals and trace elements in diabetic rats: a gender difference

**DOI:** 10.1186/1472-6823-14-72

**Published:** 2014-08-26

**Authors:** Márta Sárközy, Veronika Fekete, Gergő Szűcs, Szilvia Török, Csilla Szűcs, Judit Bárkányi, Zoltán V Varga, Imre Földesi, Csaba Csonka, Csaba Kónya, Tamás Csont, Péter Ferdinandy

**Affiliations:** 1 Cardiovascular Research Group, Department of Biochemistry, Faculty of Medicine, University of Szeged, Szeged, Hungary; 2 Pharmahungary Group, Szeged, Hungary; 3 Béres Pharmaceuticals Ltd, Budapest, Hungary; 4 Department of Pharmacology and Pharmacotherapy, Faculty of Medicine, Semmelweis University, Budapest, Hungary; 5 Department of Laboratory Medicine, Faculty of Medicine, University of Szeged, Szeged, Hungary

**Keywords:** Multivitamin, Minerals, Trace elements, Prevention, Streptozotocin, Diabetes, Gender difference

## Abstract

**Background:**

Although multivitamin products are widely used as dietary supplements to maintain health or as special medical food in certain diseases, the effects of these products were not investigated in diabetes mellitus, a major cardiovascular risk factor. Therefore, here we investigated if a preparation of different minerals, vitamins, and trace elements (MVT) for human use affects the severity of experimental diabetes.

**Methods:**

Two days old neonatal Wistar rats from both genders were injected with 100 mg/kg of streptozotocin or its vehicle to induce diabetes. At week 4, rats were fed with an MVT preparation or vehicle for 8 weeks. Well established diagnostic parameters of diabetes, i.e. fasting blood glucose and oral glucose tolerance test were performed at week 4, 8 and 12. Moreover, serum insulin and blood HbA1c were measured at week 12.

**Results:**

An impaired glucose tolerance has been found in streptozotocin-treated rats in both genders at week 4. In males, fasting blood glucose and HbA1c were significantly increased and glucose tolerance and serum insulin was decreased at week 12 in the vehicle-treated diabetic group as compared to the vehicle-treated non-diabetic group. All of the diagnostic parameters of diabetes were significantly improved by MVT treatment in male rats. In females, streptozotocin treatment resulted in a less severe prediabetic-like phenotype as only glucose tolerance and HbA1c were altered by the end of the study in the vehicle-treated diabetic group as compared to the vehicle-treated non-diabetic group. MVT treatment failed to improve the diagnostic parameters of diabetes in female streptozotocin-treated rats.

**Conclusion:**

This is the first demonstration that MVT significantly attenuates the progression of diabetes in male rats with chronic experimental diabetes. Moreover, we have confirmed that females are less sensitive to STZ-induced diabetes and MVT preparation did not show protection against prediabetic state. This may suggest a gender difference in the pathogenesis of diabetes.

## Background

The rapid increase in the prevalence of diabetes mellitus across the world gives diabetes the status of an epidemic in the 21st century [1]. In the last decades, there was an explosive increase in the number of people diagnosed with diabetes worldwide due to aging as well as increasing prevalence of obesity and physical inactivity [1-3]. The total number of people with diabetes is projected to rise from 347 million in 2008 [4] to 552 million in 2030 [5].

Not only total energy intake and macronutrients including carbohydrates, protein and fat, but also micronutrients including vitamins, minerals and trace elements have effects on the severity of diabetes mellitus. Clinical studies have shown that some individual vitamins e.g. A [6], B1 [7], B3 [8], C [9,10], D [11] and E [12], minerals e.g. calcium [13], magnesium [14] and trace elements e.g. zinc [15], chrome [16] beneficially affect the complications of diabetes mellitus. In these clinical studies, effects of individual vitamins, minerals and trace elements or combination of two or three components were investigated on diabetes. Surprisingly, there is no literature data available on the effects of multivitamin, minerals and trace elements containing preparations that can be used for human treatment in diabetes mellitus.

Regular consumption of vitamin/mineral supplements is common in developed countries [17] to maintain general health. In the United States, more than half of the adult population use dietary supplements [18,19] primarily in the form of multivitamins with or without minerals [20]. In Germany, a study in 1998 reported that 18% of men and 25% of women were regular users of multivitamins in a sample of population aged 18–79 years [21]. Moreover, MVT preparations appeared on the market as medical food for diabetics; however, no literature data supports the beneficial effect of these preparations in preclinical or clinical studies.

Therefore, here we aimed to investigate if an MVT preparation containing 26 different minerals, vitamins and vitamin-like antioxidants, as well as trace elements affects the progression of diabetes in an experimental model of diabetes in rats.

## Methods

This investigation conforms to the National Institutes of Health Guide for the Care and Use of Laboratory Animals (NIH Pub. No. 85–23, Revised 1996) and was approved by the Animal Research Ethics Committee of the University of Szeged.

Two days old neonatal male and female Wistar rats were used in this study. Lactating females with their litters were separately housed in individually ventilated cages (Sealsafe IVC system, Italy) and were maintained in a temperature-controlled room with a 12:12 h light: dark cycles for four weeks. After separation from the mother at week 4, littermates were housed in pairs under the same circumstances as mentioned above until 12 weeks of age. Standard rat chow (for ingredients see Additional file 1) and tap water were supplied ad libitum throughout the study.

### Experimental protocol

**Figure 1:**
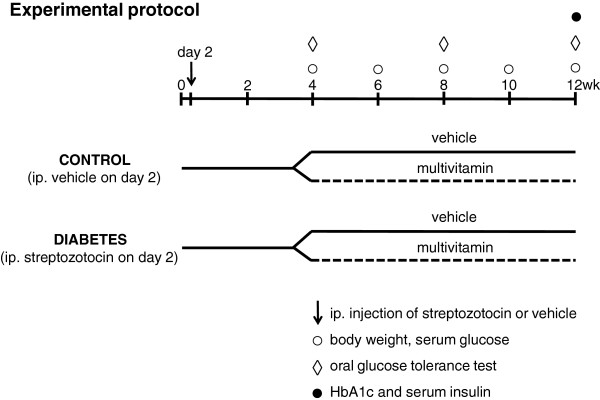
**Experimental protocol.** Two day old neonatal male and female Wistar rats were injected with 100 mg/kg of streptozotocin (STZ) (n = 69) or its vehicle (n = 38) to induce experimental diabetes mellitus. At week 4, fasting blood glucose measurement and an oral glucose tolerance test (OGTT) were performed in order to verify the development of diabetes mellitus. Both non-diabetic (n = 38) and diabetic rats (n = 40) were then fed with a mixture of vitamins, minerals, and trace elements (MVT or vehicle for eight weeks). Fasting blood glucose measurement was performed in every second week and OGTT at week 4, 8 and 12 to monitor the effect of MVT treatment on diabetes mellitus. Serum insulin and hemoglobinA1c level were measured at week 12 as well.

Two day old neonatal male and female Wistar rats were injected with 100 mg/kg of streptozotocin (STZ) (n = 69) or its vehicle (ice-cold citrate buffer) (n = 38) to induce experimental diabetes mellitus (Figure 1). Neonatal rats were kept with the mother during the lactation period until week 4. For lactating mothers, standard rat chow and tap water were supplied ad libitum throughout the study. At week 4, fasting blood glucose measurement and an oral glucose tolerance test (OGTT) were performed in order to verify the development of impaired glucose tolerance of diabetes mellitus (Figure 1) in surviving animals. In the citrate buffer-treated group, 38 animals survived (mortality rate: 0%, n = 21 male and 17 female). In the streptozotocin-treated group, 40 animals survived (mortality rate: 42%, n = 20 males and 20 females). Both non-diabetic (n = 38) and diabetic rats (n = 40) were then fed per os via gavage (1 mL/kg, 1% methylcellulose suspension) with a MVT preparation (253.3 mg/kg/day, suspended in methylcellulose) (n = 9-10) to be registered as medical food for human use (Diacomplex film-coated tablet, Béres Pharmaceuticals, Budapest, Hungary; for content see Table 1) or its vehicle (157 mg/kg/day, suspended in methylcellulose) (n = 8-11) for eight weeks (Figure 1). To conform to the human daily dose of the preparation, rat daily dose was adjusted according to the ratio of human and rat body surface areas. Fasting blood glucose measurement was performed in every second week and OGTT in every fourth week until week 12 to monitor the effect of multivitamin treatment on diabetes mellitus (Figure 1). Serum insulin and hemoglobinA1c level were measured at week 12 as well (Figure 1). To monitor the effect of diabetes mellitus on serum lipid levels, systemic inflammation, certain ion concentrations and activities of enzymes requiring vitamin cofactors, serum cholesterol, triglyceride, C-reactive protein (CRP), calcium, magnesium, phosphate and iron ion concentrations and LDH and ASAT enzyme activities were measured in the control placebo and diabetes placebo groups in both genders at week 12.

**Table 1:** Ingredients of the MVT preparation

**Active ingredients**	**Amount of ingredient/1 g product**	Daily dose*
Vitamin A_1_ (Retinol)	329 μg/g (1097 IU/g)	83.3 μg/kg/day (278 IU/kg/day)
Vitamin B_1_ (Thiamin)	2.30 mg/g	0.58 mg/kg/day
Vitamin B_2_ (Riboflavin)	2.63 mg/g	0.67 mg/kg/day
Vitamin B_3_ (Nicotinamide)	11.8 mg/g	2.99 mg/kg/day
Vitamin B_5_ (Pantothenic acid)	3.95 mg/g	1.00 mg/kg/day
Vitamin B_6_ (Pyridoxine)	3.29 mg/g	0.83 mg/kg/day
Vitamin B_12_ (Cyanocobalamin)	3 μg/g	0.76 μg/kg/day
Folic acid	197 μg/g	49.9 μg/kg/day
Biotin	99 μg/g	25.1 μg/kg/day
Vitamin D_3_ (Cholecalciferol)	3 μg/g (120 IU/g)	0.76 μg/kg/day (30.4 IU/kg/day)
Vitamin K_1_ (Phyllokinone)	26 μg/g	6.59 μg/kg/day
Rutoside	3.29 mg/g	0.83 mg/kg/day
Vitamin C	65.8 mg/g	16.7 mg/kg/day
Vitamin E	32.9 mg/g	8.33 mg/kg/day
Lutein	1.97 mg/g	0.50 mg/kg/day
Chrome	39 μg/g	9.88 μg/kg/day
Zinc	9.87 mg/g	2.50 mg/kg/day
Selenium	26 μg/g	6.59 μg/kg/day
Iron	2.63 mg/g	0.67 mg/kg/day
Iodine	66 μg/g	16.7 μg/kg/day
Manganese	0.66 mg/g	0.17 mg/kg/day
Copper	921 μg/g	233 μg/kg/day
Molybdenum	49 μg/g	12.4 μg/kg/day
Magnesium	65.8 mg/g	16.7 mg/kg/day
Calcium	132 mg/g	33.4 mg/kg/day
Phosphorus	102 mg/g	25.8 mg/kg/day

*To conform to the human daily dose of the preparation, rat daily dose was adjusted according to the ratio of human and rat body surface areas.

### Blood glucose measurements and oral glucose tolerance test (OGTT)

Rats were fasted overnight (12 h) prior to blood glucose level measurements (week 4, 6, 8, 10 and 12) and OGTTs (week 4, 8 and 12) in order to verify the development of diabetes mellitus and to monitor the effect of multivitamin treatment on diabetes. Blood samples were collected from the saphenous vein. Blood glucose levels were measured using Accucheck blood glucose monitoring systems (Roche Diagnostics Corporation, USA, Indianapolis) [22,23]. OGTT was performed as follows. After measurement of baseline glucose concentrations, glucose at 1.5 g/kg body weight was administered via oral gavage and plasma glucose levels were measured 30, 60 and 120 minutes later and area under the curve was determined [22,23].

### Hemoglobin A1c

In order to monitor the chronic effect of MVT containing preparation on diabetes mellitus, hemoglobin A1c was measured from whole venous blood with an in vitro test (Bio-Rad in2it System) according to the instructions of the manufacturer. The test is based on single wave length photometry (440 nm) to detect glycated fraction separated from the non-glycated fraction by boronate affinity chromatography.

### Measurement of serum insulin levels

To monitor the effect of MVT treatment on diabetes mellitus, serum insulin levels were measured by an enzyme immunoassay (Mercodia, Ultrasensitive Rat Insulin ELISA) in duplicates according to the manufacturer’s instructions as described [22].

### Measurement of serum cholesterol and triglyceride levels

Serum cholesterol and triglyceride levels were measured with test kits supplied by Diasys Diagnostic Systems (Holzheim, Germany) according to the instructions of the manufacturer.

### Measurement of serum CRP level as a systemic inflammatory marker

Serum CRP level was determined with a commercially available immunturbidimetric kit from Roche Diagnostics (Mannheim, Germany) according to the instructions of the manufacturer.

### Measurement of serum ion concentrations

Serum calcium, magnesium, phosphate and iron ion concentrations were measured with test kits from Roche Diagnostics (Mannheim, Germany) according to the instructions of the manufacturer.

### Measurement of serum enzyme activities

Serum ASAT and LDH activities were measured in control placebo and diabetes placebo groups with kits from Roche Diagnostics (Mannheim, Germany) according to the instructions of the manufacturer.

### Statistical analysis

Statistical analysis was performed by using Sigmaplot 12.0 for Windows (Systat Software Inc). All values are presented as mean ± SEM. Two way repeated measures ANOVA was used to determine the effect of diabetes, MVT, and the interaction of these two factors on body weight, fasting blood glucose and glucose levels during oral glucose tolerance test in the entire population as well as in males or females, respectively. Two-Way ANOVA was used to determine the effect of diabetes, MVT, and the interaction of these two factors on glucose tolerance test AUC, HbA1c and blood glucose in the entire population as well as in males or females, respectively. After ANOVA, all pairwise multiple comparison procedures with Holm-Sidak post hoc tests were used as multiple range tests. In case of serum triglyceride, cholesterol and CRP levels, ion concentrations and enzyme activities, two sample t-tests were used to determine statistical significance between groups. P ≤ 0.05 was accepted as a statistically significant difference.

## Results

**Figure 2:**
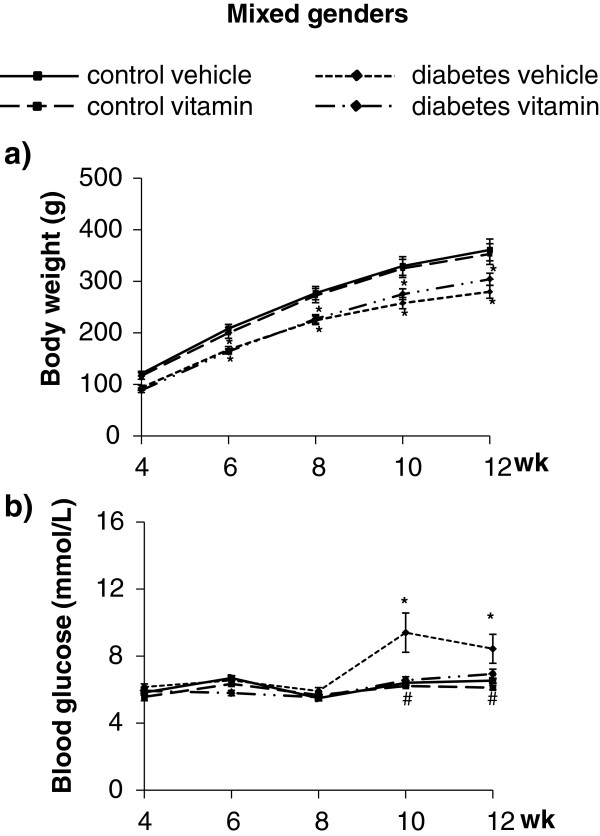
**Body weight (panel a, n = 18-20) and fasting blood glucose level (panel b, n = 18-20) in mixed genders.** Values are means ± SEM, *p < 0.05 control vs. diabetes; ^#^p ≤ 0.05 vehicle vs. vitamin in case an interaction was found between the two factors (control/diabetes and placebo/MVT).

**Figure 3:**
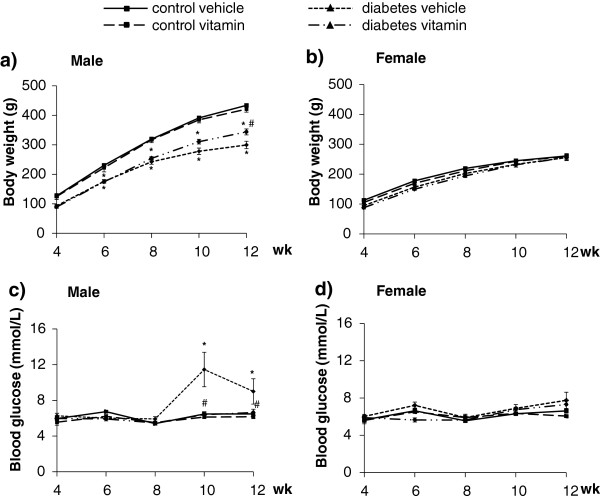
**Body weight (panel a, n = 10-11in males and panel b, n = 8-9 in females) and fasting blood glucose level (panel c, n = 10-11 in males, panel d, n = 8-9 in females).** Values are means ± SEM, *p < 0.05 control vs. diabetes; #p ≤ 0.05 vehicle vs. vitamin in case an interaction was found between the two factors (control/diabetes and placebo/MVT).

In order to verify the development of diabetes mellitus in the STZ-treated rats, concentrations of several plasma metabolites and body weight were measured at week 4 and during the treatment and follow-up period at week 6, 8, 10 and 12 (Figure 1). In the entire population of experimental animals of mixed genders, STZ-treated rats showed lower body weight from week 8 to week 12, and significantly increased fasting blood glucose at week 10 and 12 (Figure 2). In STZ-treated group, blood glucose levels were significantly decreased by MVT treatment at week 10 and 12 (Figure 2). However, male STZ-treated rats showed a significant rise in fasting blood glucose level at week 10 and 12 and a marked decrease in body weight from week 6 to the end of the follow up period (Figure 3a and c) showing the development of diabetes mellitus. Female STZ-treated rats failed to show elevated serum fasting glucose level and reduced body weight during the whole follow up period (Figure 3b and d).

**Figure 4:**
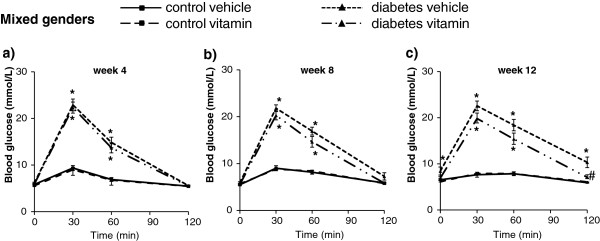
**Blood glucose in both genders at week 4, 8 and 12.** Values are means ± SEM, *p < 0.05 control vs. diabetes; n = 18-20 in each group. #p ≤ 0.05 vehicle vs. vitamin in case an interaction was found between the two factors (control/diabetes and placebo/MVT). Panel **a** demonstrates blood glucose concentration at week 4, Panel **b** at week 8 and Panel **c** at week 12.

**Figure 5:**
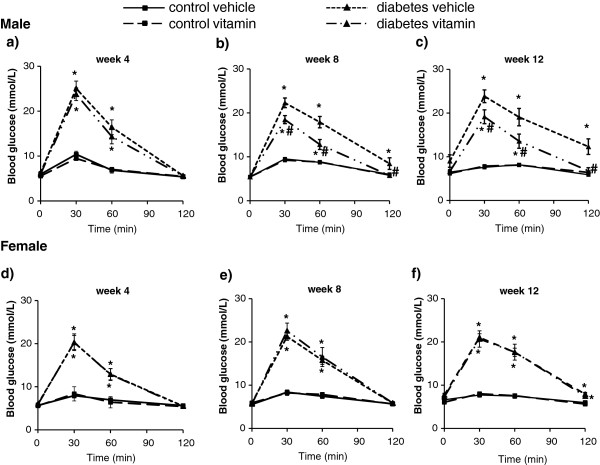
**Blood glucose in males (n = 10-11) and females (n = 8-9) at week 4, 8 and 12.** Values are means ± SEM, *p < 0.05 control vs. diabetes; #p ≤ 0.05 vehicle vs. vitamin in case an interaction was found between the two factors (control/diabetes and placebo/MVT). Panel **a** demonstrates blood glucose concentartions at week 4 in males, Panel **b** at week 8 in males, Panel **c** at week 12 in males, Panel **d** at week 4 in females, Panel **e** at week 8 in females and Panel **f** at week 12 in females.

Oral glucose tolerance test showed increased area under the curve (AUC) in mixed genders, male and female in STZ-treated rats at week 4, 8 and 12 showing impaired glucose tolerance (Figures 4 and 5 and Table 2). MVT treatment showed significant decrease in OGTT AUC values at week 12 in case of mixed genders (Table 2). Separating the genders, multivitamin treatment decreased significantly the OGTT AUC only in male STZ-treated rats at week 8 and 12 proving an anti-diabetic effect of multivitamin treatment (Table 2). However, MVT treatment did not change OGTT AUC in male control rats (Table 2). Interestingly, AUC remained unchanged by multivitamin treatment in female animals both in STZ-treated and control groups as well as at week 8 and 12 (Table 2).

**Table 2:** OGTT AUC glucose values

OGTT AUC glucose (min*mmol/L)	**Control**		Diabetes*		**Week**	**n**
	**Vehicle**	**MVT**	**Vehicle**	**MVT**		
Mixed genders	818 ± 22	821 ± 18	1615 ± 84	1539 ± 82	4	18-20
Mixed genders	895 ± 22	900 ± 21	1723 ± 76	1480 ± 86	8	18-20
Mixed genders	858 ± 13	866 ± 17	1942 ± 123	1596 ± 108^#^	12	18-20
Males	829 ± 24	846 ± 13	1754 ± 114	1613 ± 112	4	10-11
Males	940 ± 17	932 ± 24	1816 ± 126	1384 ± 74^#^	8	10-11
Males	868 ± 14	884 ± 25	2076 ± 195	1486 ± 130^#^	12	10-11
Females	808 ± 41	785 ± 37	1444 ± 102	1437 ± 118	4	8-9
Females	839 ± 38	857 ± 33	1609 ± 59	1597 ± 167	8	8-9
Females	846 ± 25	841 ± 12	1776 ± 75	1746 ± 181	12	8-9

Two Way ANOVA, *p < 0.05, Control vs. Diabetes including all groups in all time points, ^#^p ≤ 0.05 vehicle vs. vitamin in case an interaction was found between the two factors (control/diabetes and placebo/MVT).

**Figure 6:**
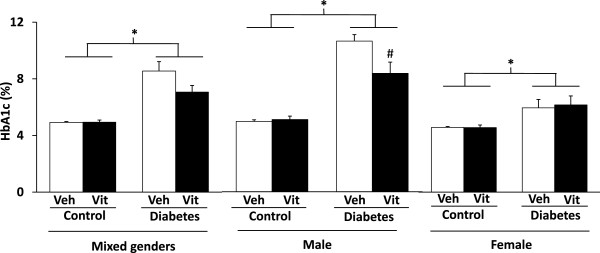
**HbA1c levels in mixed genders (n = 18-20), males (n = 10-11) and females (n = 8-9) at week 12.** Values are means ± SEM, *p < 0.05 control vs. diabetes; ^#^p < 0.05 vehicle (Veh) vs. vitamin (Vit) (p = 0.097 vehicle vs. vitamin in both genders). #p ≤ 0.05 vehicle vs. vitamin in case an interaction was found between the two factors (control/diabetes and placebo/MVT).

**Figure 7:**
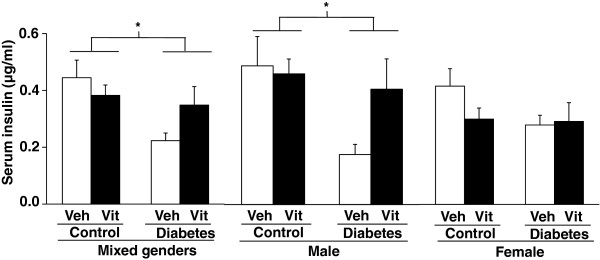
**Serum insulin levels in mixed genders (n = 18-20), males (n = 10-11) and females (n = 8-9) at week 12.** Values are means ± SEM, *p < 0.05, control vs. diabetes; ^#^p < 0.05 vehicle (Veh) vs. vitamin (Vit). No interaction was found between the two factors (control/diabetes and placebo/MVT).

HbA1c level was significantly higher in STZ-induced diabetes in mixed genders, as well as in males and females respectively as compared to controls (Figure 6) at week 12. Interestingly, multivitamin treatment has significantly reduced HbA1c level only in STZ-treated males without having any effect in STZ-treated females and control animals in both genders (Figure 6).Serum insulin concentration was significantly decreased in STZ-treated animals proving beta cell damage (Figure 7). MVT treatment showed a statistically not significant increasing tendency in STZ-treated animals (Figure 7). Separate evaluation of the data of the genders revealed that multivitamin treatment improved serum insulin level in STZ-treated males; however, serum insulin levels remained unaffected by the multivitamin treatment in control males (Figure 7). Neither diabetes mellitus, nor multivitamin treatment had a significant effect on serum insulin levels in female animals (Figure 7).

Serum triglyceride, cholesterol and CRP levels, ion concentrations including calcium, magnesium, phosphate and iron, and LDH and ASAT enzyme activities were not significantly altered in response to diabetes in either gender in our present study (Additional file 2) in contrast to some literature data [24,25].

## Discussion

Here we have shown that chronic treatment with an MVT preparation improved well established diagnostic markers of diabetes such as fasting blood glucose, HbA1c, glucose tolerance, and serum insulin levels in male diabetic rats. Furthermore, we have shown here that females developed a less severe prediabetic-like phenotype in response to neonatal STZ-treatment as only OGTT was impaired and HbA1c was increased. In the female animals, the MVT preparation failed to attenuate the progression of diabetes significantly. This is the first demonstration, that an MVT preparation attenuates the progression of experimental diabetes in males but not in females, which may suggest a gender difference in the pathogenesis of diabetes.

Regular consumption of MVT preparations as food supplements or medical food for diabetics is common in developed countries. However, surprisingly preclinical or clinical evaluation of such preparations is not available in the literature. Therefore, here we evaluated the effect of chronic treatment with an MVT preparation containing 15 vitamins and vitamin-like substances such as lutein and rutoside, 3 minerals, and 8 micro/trace elements in experimental diabetic rats. The MVT preparation showed beneficial effects on major markers of diabetes in male but not in female rats in the present study. This is the first evidence in the literature that a complex MVT preparation developed for human consumption significantly delayed progression of diabetes in an animal model. The reason why the MVT preparation was ineffective in female rats is not known, however, it should be emphasized that female rats developed only an impaired glucose tolerant state characterized by an increased OGTT AUC and HbA1c levels in the present study. It is well known that the progression of diabetes occurs at a later age and becomes milder in females compared to age-matched males in rodent models of diabetes (see for review [26]). One may speculate that this could be one of the reasons why the MVT preparation used in our present study was unable to improve the biochemical markers of early diabetes significantly.

In our present study, neonatal STZ treatment in rats resulted in a condition that was more similar to T1DM than to T2DM. However, it has been shown in the literature that neonatal rats injected with streptozotocin developed acute diabetes followed by a spontaneous remission due to some pancreatic β-cell regeneration. Therefore, there is no total insulin deficiency, but a decreased serum insulin concentration in STZ-treated animals [27-29]. Our present study is in accordance with these literature data, because serum insulin concentration was significantly lower in males and mixed genders in STZ-treated animals at week 12 (Figure 7). The literature is limited on the effect of different individual minerals and vitamins or related gender differences on pancreatic beta cell regeneration in diabetes. However, there are some well-known vitamins and minerals including vitamin B3 and D as well as calcium ion that have been previously linked to β-cell regeneration in diabetes. Vitamin B3 has been shown to stimulate β-cell regeneration in partially pancreatectomized rats [30,31], lengthen the “honeymoon” period in type 1 diabetic patients [30,32], and improve insulin secretion from patients at high risk of developing type 1 diabetes [30,33]. Nicotinamide also protects β-cells from streptozotocin-induced damage in rodents through a suggested mechanism involving inhibition of PARP [30,34-36]. Recently, vitamin D is thought to have beneficial effects on pancreatic beta-cell dysfunction possibly via both vitamin D receptor-dependent and -independent actions in diabetic patients of both genders [37]. In addition, Ca^2+^ depletion has been shown to decrease β-cell proliferation and increase β-cell death by apoptosis [38]. These studies, in agreement with our present findings, may suggest that the multivitamin preparation beneficially affects β-cell regeneration in male rats in our present study; however, this potential effect should be investigated in further studies.

In different experimental diabetes models and clinical studies involving limited number of patients, several data are available on the effect of individual vitamins, minerals, trace elements, or the combination of limited number of them. The individual components of the MVT preparation investigated in the present study were selected by the manufacturer on the basis of their preclinical and clinical data in different diabetic animal models or patient populations. Daily doses of all components of the preparation were set below the human upper safe level [39].

Effects of individual vitamins on diabetes and/or its complications have been investigated in a number of preclinical and clinical trials. It was demonstrated that a 2-week administration of vitamin B1 in a high dose (0.2% thiamin in drinking water) prevents diabetes-induced cardiac fibrosis without reducing the blood glucose level in male diabetic rats [7]. A randomized double-blind vehicle-controlled clinical pilot study recruiting 40 type 2 diabetics with microalbuminuria demonstrated that a high-dose vitamin B1 therapy (300 mg/day) for 3 months produced a regression of urinary albumin excretion without any effect on plasma glucose or HbA1c levels [40]. Vitamin D deficiency is a known risk factor of diabetes mellitus. A double-blind parallel group vehicle-controlled randomized trial involving 87 type 2 diabetics reported that a single large dose (100,000 IU) of vitamin D2 improved endothelial function 8 weeks after the administration, however, HbA1c and HOMA-IS were unaffected by vitamin D2 therapy [41]. Additionally, another randomized, vehicle-controlled clinical trial with 81 participants showed that vitamin D3 supplementation (4000 IU) for 6 months significantly improved insulin sensitivity and fasting insulin level in type 2 diabetic women [11].

A number of preclinical and clinical trials have investigated the effects of individual minerals on diabetes and/or its complications. A randomized controlled, single-blinded trial with 31 patients demonstrated that oral calcium supplementation (1500 mg/day) for 2 months improves insulin sensitivity in patients with type 2 diabetes and hypertension, however, both fasting blood glucose and HbA1c levels were unaffected by the calcium supplementation [42]. Furthermore, it was shown that dietary calcium supplementation (600 mg/day) for 3 months significantly reduced vascular resistance and induced partial regression of left ventricular hypertrophy in hypertensive non-insulin-dependent diabetic Afro-Americans [13]. A clinical randomized double-blind vehicle-controlled trial recruiting 63 type 2 diabetic patients with hypomagnesemia receiving glibenclamide has shown that supplementation of magnesium (2.5 g MgCl_2_/day) for 4 months improved HbA1c, fasting glucose as well as insulin levels [43]. Another double-blind vehicle-controlled clinical trial enrolling 82 diabetic hypertensive adults with hypomagnesemia receiving captopril demonstrated that oral magnesium supplementation (2.5 g MgCl_2_/day) for 4 months significantly reduced fasting plasma glucose as well as HbA1c levels, systolic and diastolic blood pressure [14]. In contrast, a randomized clinical study involving 97 patients showed that chronic supplementation of magnesium (300 mg/day) for 5 years attenuated the evolution of polyneuropathy in type 1 diabetics with magnesium deficiency without reducing HbA1c level [44].

Limited number of clinical data on some individual trace elements has shown beneficial effects on diabetes and its complications. A clinical pilot study involving 22 patients demonstrated that zinc supplementation (30 mg/day) for 3 months decreased lipid peroxidation in type 1 diabetes mellitus [45]. A prospective double-blind vehicle-controlled crossover study involving 30 participants demonstrated that supplementation of chromium for 2 months significantly reduced serum triglyceride level in type 2 diabetic patients without any effect on serum glucose level [46]. In contrast, another randomized clinical study involving 180 patients with type 2 diabetes showed that chromium administration (1000 μg/day) for 4 months had beneficial effects on HbA1c, glucose, insulin, and cholesterol variables [16].

Limited data on combinations of few numbers of vitamins, minerals and trace elements have shown beneficial effects on diabetes and/or its cardiovascular complications. A randomized, double-blind, vehicle-controlled clinical trial showed that a combination of 200 mg magnesium, 30 mg zinc, 200 mg vitamin C, and 100 IU vitamin E significantly improved glomerular function [6], blood pressure [47] and increased HDL-c and apo A1 level [47] without beneficially affecting serum glucose and HbA1c levels in 69 patients with type 2 diabetes mellitus after 3 months daily treatment. A randomized clinical trial enrolling 64 children with recent onset of type 1 diabetes (IMDIAB IX) demonstrated that implementation of insulin therapy with vitamin B3 (25 mg/kg body weight) alone or in combination with vitamin E (15 mg/kg body weight) for 2 years preserved baseline C-peptide secretion without any effect on HbA1c level [48]. In contrast, a vehicle-controlled double-blind randomized clinical trial involving 348 participants has reported that statin therapy co-supplemented with biotin (2 mg/day) and chromium (600 μg/day) for 3 months has significantly decreased serum glucose, HbA1c, LDL-cholesterol, total cholesterol, and VLDL-cholesterol levels in type 2 diabetic patients [49]. In many of the abovementioned studies [6,11,40,41,45,47,49], some of the individual vitamins, minerals and trace elements were used in a daily dose above the upper safe level [39]. A pilot clinical study in Sri Lanka involving 96 patients demonstrated that a 15-component MVT preparation significantly reduced serum glucose and lipid levels in adult diabetic patients after 4 months of supplementation [36], however, the daily dose of 3 components of this preparation was above the upper safe level and no gender difference was investigated [50].

The limitation of the present study is that it does not provide evidence on the mechanism of the effect of the MVT preparation and the individual contribution of the 26 components to the anti-diabetic effect of the preparation. The potential interactions of these components and their combined effect rather than a single component could be responsible for the beneficial effects of the MVT preparation on the severity of diabetes. However, investigation of the effects of each components and their different variation of combinations was out of the scope of the present study.

## Conclusion

Although MVT preparations are widely used by diabetics, our present study is the first demonstration that a MVT preparation attenuates the progression of experimental diabetes. Moreover, it seems that there is a gender difference in the development of diabetes and the anti-diabetic effect to MVT treatment. Further studies are needed to optimize the composition and to elucidate the efficacy, safety and the mechanism of the effect of MVT preparations in diabetics.

## Competing interests

Béres Pharmaceuticals Ltd., Pharmahungary Group and University of Szeged had a consortial grant funded by the National Development Agency (MED_FOOD TECH_08-A1-2008-0275) to develop MVT preparations for diabetic patients, Béres Pharmaceuticals Ltd. was the leader of this consortial project. C.S., J. B. and C. K. are employed by Béres Pharmaceuticals Ltd. P.F. is the owner and T.C. and C.C. are involved in the management of Pharmahungary Group, a pharmaceutical/biotechnological company.

## Authors’ contributions

CK, TC and PF conception and design of research; MS, VF, GS, TS, CS, JB, ZVV and IF performed experiments; MS, VF, GS, TS, CS, JB and CC analysed data; MS, VF, GS, JB and TC interpreted results of experiments; MS prepared figures; MS, TC and PF drafted manuscript; MS, TC and PF edited and revised manuscript; MS, VF, GS, TS, CS, JB, ZVV, IF, CC, CK, TC and PF approved final version of manuscript. All authors read and approved the final manuscript.

## Pre-publication history

The pre-publication history for this paper can be accessed here:

http://www.biomedcentral.com/1472-6823/14/72/prepub

## Supplementary Material

Additional file 1 Ingredients of the standard rat chow. Click here for file

Additional file 2 Effect of diabetes mellitus on serum triglyceride, cholesterol and CRP levels, calcium, magnesium, phosphate and iron concentrations, LDH and ASAT enzyme activities in both genders. Click here for file
