# Supplementary material for: Anti-diabetic effect of a preparation of vitamins, minerals and trace elements in diabetic rats: a gender difference
Source: BMC Endocr Disord. 2014 Aug 26;14:72. doi: 10.1186/1472-6823-14-72 (PMC4170941; doi:10.1186/1472-6823-14-72)
Supplement: Additional file 1 — Ingredients of the standard rat chow. [file 1472-6823-14-72-S1.docx]

Additional material 1

Ingredients of the standard rat chow

| **Ingredients** | **Amount of ingredient** |
| --- | --- |
| Dry matter | 89 % |
| Raw protein | 19.9 % |
| Raw fat | 3.6 % |
| Raw fibers | 4.6 % |
| Lysine | 0.935 % |
| Methionine | 0.373 % |
| Cysteine | 0.377 % |
| Threonine | 0.771 % |
| Tryptophan | 0.244 % |
| Vitamin A_1_ (Retinol) | 12000 IU/kg |
| Vitamin B_1_ (Thiamin) | 1.5 mg/kg |
| Vitamin B_2_ (Riboflavin) | 5.0 mg/kg |
| Vitamin B_5_ (Pantothenic acid) | 14.0 mg/kg |
| Vitamin B_6_ (Pyridoxal phosphate) | 2.5 mg/kg |
| Vitamin B_12_ (Cyanocobalamin) | 0.02 mg/kg |
| Folic acid | 0.5 mg/kg |
| Biotin | 0.105 mg/kg |
| Vitamin D_3_ (Cholecalciferol) | 1200 IU/kg |
| Vitamin K_1_ (Phyllokinone) | 1.00 mg/kg |
| Vitamin E | 40 IU/kg |
| Zinc | 47.71 mg/kg |
| Selenium | 0.10 mg/kg |
| Iodine | 1.000 mg/kg |
| Iron | 111.02 mg/kg |
| Manganese | 48.47 mg/kg |
| Copper | 10.65 mg/kg |
| Cobalt | 1.00 mg/kg |
| Sodium | 0.172 % |
| Magnesium | 0.068 % |
| Calcium | 0.795 % |
| Phosphorus | 0.594 % |
