# Supplementary material for: Anti-diabetic effect of a preparation of vitamins, minerals and trace elements in diabetic rats: a gender difference
Source: BMC Endocr Disord. 2014 Aug 26;14:72. doi: 10.1186/1472-6823-14-72 (PMC4170941; doi:10.1186/1472-6823-14-72)
Supplement: Additional file 2 — Effect of diabetes mellitus on serum triglyceride, cholesterol and CRP levels, calcium, magnesium, phosphate and iron concentrations, LDH and ASAT enzyme activities in both genders. [file 1472-6823-14-72-S2.docx]

Additional material 2

Effect of diabetes mellitus on serum triglyceride, cholesterol and CRP levels, calcium, magnesium, phosphate and iron concentrations, LDH and ASAT enzyme activities in both genders

|  | **Males** | | |
| --- | --- | --- | --- |
|  | Control Placebo | Diabetes Placebo | t-test |
| n | 4 | 4 | p-value |
| Triglyceride (mmol/L) | 0.93 ± 0.17 | 0.79 ± 0.15 | 0.579 |
| Cholesterol (mmol/L) | 3.16 ± 0.55 | 1.81 ± 0.47 | 0.136 |
| CRP (mg/L) | 25.7 ± 0.43 | 25.6 ± 0.17 | 0.834 |
| Calcium (mmol/L) | 2.32 ± 0.17 | 2.18 ± 0.24 | 0.656 |
| Magnesium (mmol/L) | 0.85 ± 0.06 | 0.71 ± 0.08 | 0.222 |
| Phosphate (mmol/L) | 1.87 ± 0.15 | 1.33 ± 0.18 | 0.068 |
| Iron (mmol/L) | 28.0 ± 3.2 | 24.5 ± 4.8 | 0.556 |
| LDH (IU/L) | 766 ± 272 | 497 ± 162 | 0.427 |
| ASAT (IU/L) | 85.8 ± 16.5 | 63.0 ± 11.1 | 0.296 |

|  | **Females** | | |
| --- | --- | --- | --- |
|  | Control Placebo | Diabetes Placebo | t-test |
| n | 3 | 4 | p-value |
| Triglyceride (mmol/L) | 0.64 ± 0.07 | 1.11 ± 0.17 | 0.074 |
| Cholesterol (mmol/L) | 2.14 ± 1.32 | 2.64 ± 0.70 | 0.731 |
| CRP (mg/L) | 26.2 ± 0.43 | 26.05 ± 0.68 | 0.816 |
| Calcium (mmol/L) | 1.55 ± 0.48 | 1.94 ± 0.18 | 0.434 |
| Magnesium (mmol/L) | 0.56 ± 0.11 | 0.70 ± 0.07 | 0.306 |
| Phosphate (mmol/L) | 1.1 ± 0.35 | 1.44 ± 0.18 | 0.395 |
| Iron (mmol/L) | 18.2 ± 3.6 | 22.3 ± 3.7 | 0.474 |
| LDH (IU/L) | 493 ± 232 | 560 ± 275 | 0.867 |
| ASAT (IU/L) | 53.7 ± 12.4 | 71.8 ± 21.4 | 0.539 |
